# Endovascular Transvenous versus Open Femoropopliteal Bypass

**DOI:** 10.3390/medicina60050777

**Published:** 2024-05-08

**Authors:** Roberts Rumba, Dainis Krievins, Natalija Ezite, Aigars Lacis, Ludovic Mouttet, Anda L. Vavere, Christopher K. Zarins

**Affiliations:** 1 Vascular Surgery Department, Pauls Stradins Clinical University Hospital, 1002 Riga, Latvia; roberts.rumba@stradini.lv (R.R.);; 2 Diagnostic Radiology, Diagnostic and Interventional Radiology Centre, Pauls Stradins Clinical University Hospital, 1002 Riga, Latvia; 3 Department of Surgical and Interventional Sciences, Faculty of Medicine and Health Sciences, McGill University, Montreal, QC H3G 2M1, Canada; 4 Faculty of Medicine, University of Latvia, 1004 Riga, Latvia; 5 Department of Surgery, Stanford University Medical Center, Stanford, CA 95305, USA

**Keywords:** transvenous femoropopliteal bypass, open bypass, prosthetic graft, autologous vein, patency data

## Abstract

*Background and Objectives*: Lower extremity arterial disease is one of the most prevalent manifestations of atherosclerosis. The results from numerous studies regarding the best revascularization method of an occluded superficial femoral artery have been conflicting. The aim of this study was to compare the patency of transvenous endovascular with open femoropopliteal bypass, both with vein and prosthetic grafts. To our knowledge, a direct patency comparison between transvenous endovascular and open femoropopliteal bypass has not been published. This could help elucidate which method is preferable and in which cases. *Materials and Methods*: Patients with complex TASC-C and D SFA lesions were offered endovascular transvenous or open bypass. A total of 384 consecutive patients with PAD requiring surgical treatment were evaluated for inclusion in this study. Three-year follow-up data were collected for 52 endovascular procedures, 80 prosthetic grafts, and 44 venous bypass surgeries. Bypass patency was investigated by Duplex US every 6 months. Kaplan–Meier plots were used to analyze primary, primary-assisted, and secondary patency for endovascular transvenous, autovenous, and prosthetic bypasses. *Results*: Primary, primary-assisted, and secondary patency in venous group at 3 years was 70.5%, 77.3%, and 77.3%, respectively. In the endovascular transvenous group, primary, primary-assisted, and secondary patency at 3 years was 46.2%, 69.2%, and 76.9%, respectively. The lowest patency rates at 3 years were noted in the prosthetic graft group with 22.5% primary, 26.6% primary-assisted, and 28.2% secondary patency. *Conclusions*: The saphenous vein is the best graft to perform in above-the-knee femoropopliteal bypass. Transvenous endovascular bypass is a viable option with comparable primary-assisted and secondary patency. Primary patency is substantially lower for endovascular transvenous compared to venous bypass. Patients treated with endovascular transvenous bypass will require a significant number of secondary procedures to provide optimal patency. Prosthetic grafts should only be used if no other option for bypass is available.

## 1. Introduction

Lower extremity arterial disease is one of the most prevalent manifestations of atherosclerosis, which effects both the lifespan and quality of life of more than 230 million people worldwide [1]. Despite the fact that more than 50% of patients with peripheral arterial disease (PAD) are asymptomatic, it increases the risk of all-cause mortality by 60% and cardiovascular death by 96% [2]. Approximately 11% of all diagnosed cases with PAD develop chronic limb-threatening ischemia, a condition which carries an annual combined mortality and major amputation incidence of 20% [1]. Due to the increasing prevalence of type 2 diabetes and persistence of smoking, the burden of chronic limb-threatening ischemia (CLTI) is expected to rise along with the need for effective revascularization procedures [3]. Traditionally, patients with complex TASC II C and D superficial femoral artery (SFA) lesions were treated with open surgery, but, due to several advances in technology and experience, endovascular treatments have become increasing more utilized. Several trials have compared an endovascular first approach with open surgery treatment to asses which option leads to superior outcomes in different clinical situations. Studies have found largely conflicting evidence on which method is superior to revascularize total SFA occlusions. Both non-randomized and randomized trials have indicated the superiority of open surgery over endovascular treatment [4,5], and vice versa [3,6,7], especially if the autologous saphenous vein is not available for bypass. Due to its less invasive nature, endovascular treatment generally provides a lower morbidity, periprocedural mortality, and length of hospital stay [6,7], whereas open surgery provides lower rates of major adverse limb events and death in the long term [4]. However, not every patient is eligible for open surgery. To overcome this limitation and to expand the spectrum of patients to whom the revascularization of long complex SFA occlusions can be provided, the transvenous endovascular femoropopliteal bypass procedure was developed (named the Detour procedure by company PQ Bypass). This technique has been extensively reported with regard to its safety [8,9] and 3-year graft patency [10]. To our knowledge, a direct patency comparison between transvenous endovascular and open femoropopliteal bypass has not been published. We, therefore, performed a study comparing the patency data of open and endovascular bypasses in our vascular surgery center.

## 2. Materials and Methods

**Figure 1:**
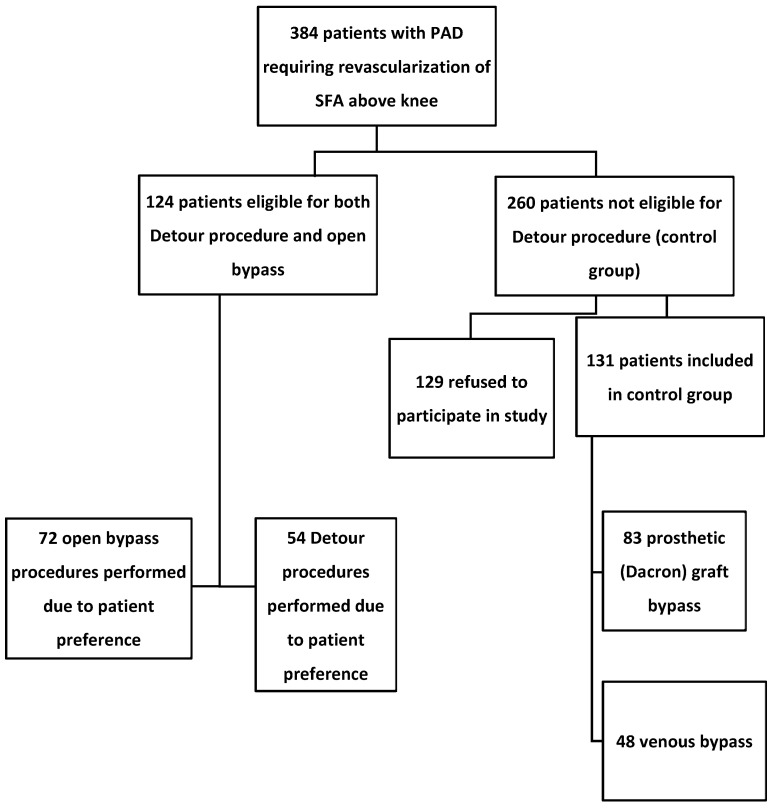
Flow-chart depicting patient treatment and selection protocol. PAD, peripheral artery disease; SFA, superficial femoral artery.

This was a single-center study which took place from 2015 until 2020 in Pauls Stradins Clinical University Hospital, Riga, Latvia. The aim of this study was to compare clinical improvements, patency, and safety of endovascular transvenous above-the-knee femoropopliteal bypass with conventional open bypass with either prosthetic grafts or autologous saphenous vein. During study, a total of 384 consecutive patients with PAD requiring surgical treatment were evaluated for inclusion in this study. A flow-chart illustrating patient selection is depicted in Figure 1. After eligibility assessment for Detour procedure, this treatment was discussed with and offered to 124 patients, of whom 52 patients preferred this procedure over open bypass. Two patients had bilateral total SFA occlusions; hence, 54 procedures were performed. In efforts to eliminate selection bias, Detour procedure was only offered to patients who were also suitable for open above-the-knee femoropopliteal bypass. The remaining 260 patients received open femoropopliteal above knee bypass, and were offered to participate in this follow-up patency study as control subjects. Those 131 that did agree to participate were included and formed two control groups—83 in prosthetic graft and 48 in venous bypass group. During the first year, two patients in prosthetic graft group and three in venous bypass group did not attend follow-up, and one patient from transvenous group died after 8 months with unrelated cause. One patient was lost to follow-up after 24 months in each of control group and one in the endovascular bypass group. Therefore, 3-year follow-up data are available for 52 endovascular procedures, 80 prosthetic grafts, and 44 venous bypass surgeries. Inclusion criteria for endovascular transvenous femoropopliteal bypass were SFA TASC C or D lesions with a patent popliteal and at least one crural artery, Rutherford class 3–5, and an ABI of 0.7 or less. Preoperative Duplex ultrasound was performed to evaluate the diameter of the popliteal vein (>10 mm considered eligible for procedure). Patients with stage 4–5 chronic kidney disease, history of deep venous thrombosis (DVT), previous major amputation in index or non-index leg, and Rutherford categories 0–2 and 6 were excluded from this study. Ethics committee approved this study and written informed consent was obtained from all the participants.

All endovascular procedures were performed by the same team of vascular surgeons and interventional radiologists, whereas open surgeries were performed by vascular surgeons in the same department. Notably, the technique of open femoropopliteal bypass in our department is highly standardized due to a very strong conservative school of training. None of the open surgeries were in situ bypasses.

Description of endovascular transvenous bypass technique has been published previously [10,11]. In summary, all patients receive 5000 units of heparin with a target activated clotting time of 250–300 s. Arterial access is established using contralateral common femoral artery, followed by aortic bifurcation crossover to place PQ Crossing device (PQ Bypass, Milpitas, CA, USA) just proximal to SFA occlusion. Ipsilateral crural or muscle veins are accessed using ultrasound guidance to introduce PQ Snare device in the femoral vein (PQ Bypass, Milpitas, CA, USA) directly opposing PQ Crossing device, which contains an X-ray marker. This is used to guide C-arm rotation and to position the Crossing device towards the PQ Snare in the femoral vein. A needle is then deployed through the wall of SFA, femoral vein, and into the PQ Snare device, which contains a basket for snaring the guidewire. This ensures a through-and-through access. After balloon dilation of proximal anastomosis, PQ Crossing device is pulled into femoral vein and advanced distally to a level of patent artery. Using the spring-loaded delivery system, needle is used to penetrate vein wall and deliver guidewire into patent artery lumen using X-ray markers. Distal anastomosis is established using balloon dilation, and the final step is TORUS stent graft (PQ Bypass, Milpitas, CA, USA; self-expanding nitinol wire encapsulated in ePTFE) deployment in distal-to-proximal fashion. Two stent grafts were used for most patients (59.3%), whereas three were necessary in 38.8% of cases, and one patient required four devices.

Open femoropopliteal above-the-knee bypass technique has been extensively described and illustrated numerous times and is beyond the scope of this article. For control group patients, either reversed autologous great saphenous vein or Intergard Silver knitted Dacron (Getinge, Gothenburg, Sweden) prosthetic grafts were used. No hand veins, small saphenous veins, or spliced veins were used for bypass.

Following all procedures and open surgeries, patients were invited for control visits in study center with 6-month intervals for a total follow-up of 3 years. During visits, Duplex ultrasound was performed to assess graft patency, inflow and outflow vessels, and venous system in patients with transvenous grafts. Due to a stent-graft placement, the transvenous bypass group received a dual antiplatelet treatment (Aspirin 100 mg (G.L. Pharma GmbH, Lannach, Austria) + Clopidogrel 75 mg (Sanofi Winthrop Industrie, Ambarès-et-Lagrave, France)) throughout the 3-year follow-up, except for those on anticoagulants because of other indications. Patients in control groups received either standard aspirin monotherapy, dual antiplatelet therapy, or anticoagulants if indicated by coronary artery disease or arrythmia, respectively. Antiplatelet therapy was temporarily stopped if clinically significant bleeding occurred and was reinitiated as soon as possible.

We defined primary patency as time in months from index procedure/surgery to any secondary procedure in case of significant stent graft or bypass stenosis. If significant stenosis was diagnosed and an intervention was performed to correct it, this period was defined as primary-assisted patency time. If, however, a full stent graft or bypass occlusion occurred and was treated with thrombectomy, this additional patency time was summed up to form secondary patency.

IBM SPSS Statistics 29 was used for statistical analysis. Descriptive statistics, including means, standard deviations, and frequencies, were used to characterize the demographic and baseline data. For continuous variables, independent-sample *t*-test and Mann–Whitney U test were used to assess statistical significance of differences. For categorical variables, Chi-square test was utilized. Graft patency was analyzed using Kaplan–Meier plots. Log-rank test was used to compare differences in patency between study groups. In efforts to identify factors that influence graft patency, we used Cox proportional hazards analysis. *p* value < 0.05 was considered significant.

## 3. Results

### 3.1. Study Population—Endovascular Transvenous Group

In the transvenous endovascular group, the mean age of the patients was 64.9 years (SD = 8.3). The majority of the patients were male (92.3%). Before surgery, the mean ABI was 0.62 (SD = 0.16). The mean body mass index was 27.5 (SD = 5.1). Rutherford class 3 patients comprised the majority of the endovascular group (84.6%). Typical comorbidities of this population were prevalent—arterial hypertension (88.4%, *n* = 46), smoking (83.3%, *n* = 45), and coronary artery disease (28.8%, *n* = 15).

### 3.2. Study Population—Control Group

The mean age of the patients in the control group was 70.6 (SD = 6.6); the oldest patient was 88 and the youngest 56. More male patients were present in the control group (70.4%). The mean baseline ABI in the control group was 0.56 (ranging from 0.3–0.7). Table 1 summarizes the study population data.

### 3.3. Clinical Improvements

The majority of patients in both groups experienced significant improvements in clinical symptoms immediately after surgery, as was expected following the successful revascularization of an occluded SFA. A reduction of at least one Rutherford class was observed in 96% of patients in the endovascular transvenous group and 95.2% of patients in the open surgery group, evaluated one month after surgery. A year later, 92.2% of patients in the endo group and 92.7% in the open surgery group were still at least one Rutherford class lower than the baseline. Clinical improvements persisted for two years in 85.4% of cases in the endo group and 87.9% in the open surgery group. At the 3-year follow-up, endovascular transvenous treatment provided clinical improvement to 73.2% of patients and open surgery to 76.6%. The differences between groups were not statistically significant.

### 3.4. Primary Patency

**Figure 2:**
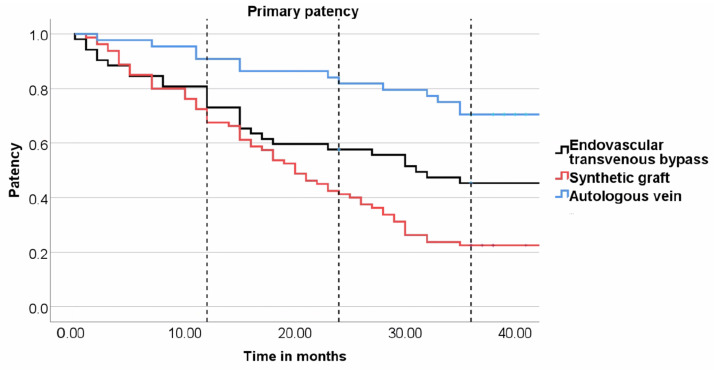
Kaplan–Meier plot for primary patency. Dotted lines indicate 12-month, 24-month, and 36-month follow-up. Venous bypass (blue line), endovascular transvenous bypass (black line), and prosthetic graft bypass (red line).

Significant differences in primary patency were noted early in the follow-up. At 12 months, vein bypass performed better than both endovascular and prosthetic grafts in terms of primary patency—90.9% for venous, 79.6% for endovascular transvenous, and 72.5% for prosthetic bypass. The difference reached statistical significance for venous versus prosthetic grafts (*p* = 0.015), but there was also a trend towards significance for venous versus endovascular (*p* = 0.095). After 24 months, venous bypass (84.1%) outperformed both endovascular (58.5%, *p* = 0.006) and prosthetic grafts (42.5%, *p* < 0.001) in primary patency. Three years after the index procedure, primary patency dropped to 22.5% for prosthetic grafts and 46.2% for endovascular transvenous, and remained relatively high (70.5%) for autologous vein bypasses. See Figure 2.

### 3.5. Primary-Assisted Patency

**Figure 3:**
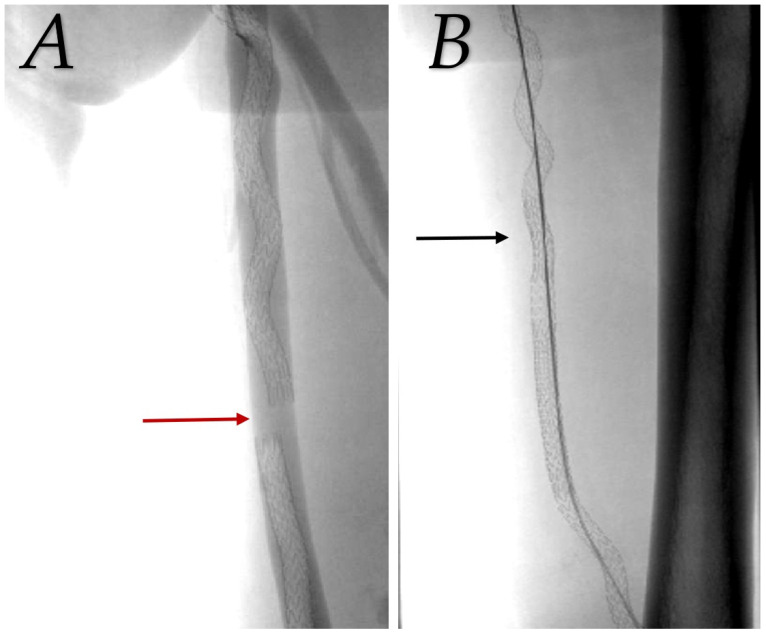
Salvage procedure to bridge detached stent grafts. (**A**) Red arrow indicates a gap between migrated covered stents in femoral vein. (**B**) Additional stent graft (black arrow) used to bridge detached segment and treat iatrogenic arteriovenous fistula.

**Figure 4:**
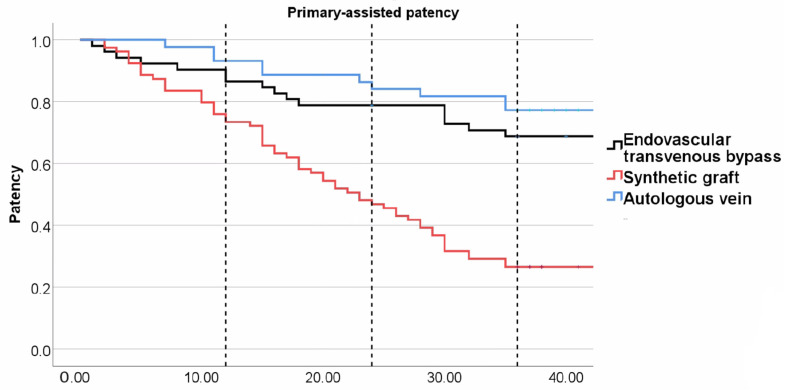
Kaplan–Meier plot for primary-assisted patency. Dotted lines indicate 12-month, 24-month, and 36-month follow-up. Venous bypass (blue line), endovascular transvenous bypass (black line), and prosthetic graft bypass (red line).

During the first year, five angioplasties were performed for endovascular transvenous bypasses, three for prosthetic grafts, and one for venous bypasses. We had two cases of stent-graft migration due to an insufficient overlap in the endovascular group, which required a salvage procedure to bridge migrated graft components one day and one month after primary procedure (see Figure 3). Primary-assisted patency was 88.9% for transvenous endovascular, 75.9% for prosthetic grafts, and 93.2% for venous bypasses after 12 months (*p* = 0.013 for prosthetic versus venous bypass). To maintain graft patency, six additional angioplasties were performed during year two in the endovascular group, two in the prosthetic graft, and none in the vein group. At the 24-month visit, both endovascular (79.2%, *p* = 0.002) and venous bypasses (86.4%, *p* < 0.001) were significantly more patent than prosthetic grafts (48.1%) with regard to primary-assisted patency. This was even more pronounced at 36 months (see Figure 4).

### 3.6. Secondary Patency

**Figure 5:**
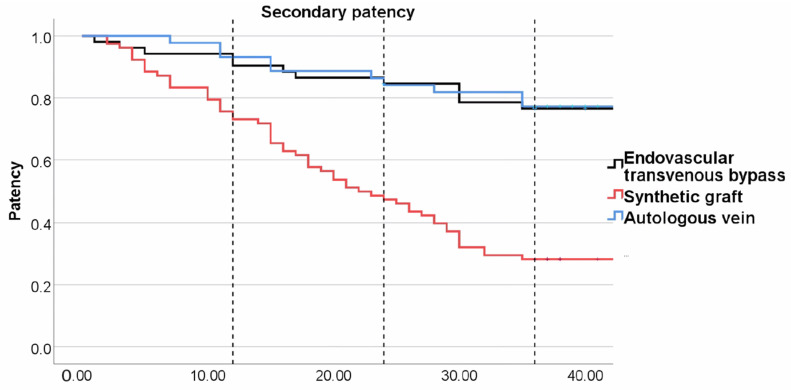
Kaplan–Meier plot for secondary patency. Dotted lines indicate 12-month, 24-month, and 36-month follow-up. Venous bypass (blue line), endovascular transvenous bypass (black line), and prosthetic graft bypass (red line).

Secondary patency during the first 12 months was significantly better for the endovascular transvenous group (92.6%, *p* = 0.21) and venous group (93.2%, *p* = 0.012) compared to prosthetic grafts (75.6%). This trend persisted during the second year, with a small decrease in secondary patency in the endovascular transvenous (86.8%) and venous bypass group (86.4%). Prosthetic grafts, however, occluded significantly more often (48.7% secondary patency at 24 months). At the end of the study, there was not a significant difference in secondary patency between the venous (77.3%, *p* = 908) and transvenous endovascular groups (76.9%). Patency was notably lower in the prosthetic graft group at 28.2% (Figure 5). Table 2 summarizes the patency data with *p* values where significant.

### 3.7. Cox Proportional Hazards

We performed a Cox proportional hazards analysis to identify if the patient age, Rutherford category, or number of patent outflow vessels (0–3) had a significant effect on the bypass patency time. The analysis was performed separately for primary, primary-assisted, and secondary patency. None of the aforementioned variables had a statistically significant influence on bypass patency rates.

## 4. Discussion

Numerous trials have been performed to answer the question as to which technique is preferable in order to revascularize complex SFA lesions, mainly, chronic total occlusions, and for which patient. The results of these studies have been conflicting. The conventional approach was to use endovascular techniques for more simple lesions and open surgery in complex TASC C and D cases. Data from the BASIL-1 trial indicated the superior amputation-free survival and overall survival in the open surgery group compared to angioplasty in patients with CLTI. These data have since been criticized for having a low generalizability, since only 30% of patients in this trial were eligible for randomization [12].

The approach to SFA revascularization has changed significantly with the development of more advanced and effective endovascular devices tailored to different lesions and procedural stages. There are currently many proponents of the “endovascular first” approach, stating higher patency rates and lower major adverse limb events compared to prosthetic grafts at 24 months [6]. In a large randomized BASIL-2 trial, the ‘’endovascular first’’ strategy was even shown to be superior than venous bypass for infra-popliteal revascularization in patients with CLTI [3]. On the other hand, if endovascular revascularization is unsuccessful, the results of the subsequent open surgery are shown to be worse than with primary open revascularization [13]. One of the factors that needs to be considered is the patient’s overall health status and comorbidities. A recent meta-analysis reported a lower 30-day mortality in the endovascular revascularization group compared to open surgery, in both the cohort data (OR = 0.79, CI: 0.67–0.94) and four randomized controlled trials (RCTs) (OR = 0.56, CI:0.33–0.94) [14]. No significant difference was noted in 30-day major amputation rates or in overall survival in RCTs. The endovascular group showed lower rates of wound complications as expected, but higher rates of reintervention in the combined cohort data but not in RCTs. The risk of failure of primary (HR: 1.23, 95% CI: 1.02–1.49) and secondary (HR: 2.05, 95% CI: 1.41–3.00) patency was higher in the endovascular group in the pooled RCT data [14]. The aforementioned meta-analysis concluded that heterogeneity in results was present and could be explained by regional differences. Efforts to assess our local results are thus relevant.

It is important to emphasize that “open surgery” is not a homogenous group. Numerous studies, including a recent systematic review and meta-analysis, have concluded that venous bypass has higher primary, primary-assisted, and secondary patency compared to prosthetic grafts [15] for above-the-knee revascularization. This is in accordance with the Cochrane review data, which concluded that autologous vein bypass improves primary patency by 60 months compared to prosthetic grafts. Similarly, we have also observed significantly higher patency rates in the venous bypass group compared to prosthetic bypasses in our study. The Cochrane review did not find a significant difference between Dacron and PTFE grafts in terms of primary patency. Only low-quality evidence suggested the superiority of Dacron grafts over PTFE in secondary patency [16]. Despite higher patency rates for venous bypasses, there was not a significant difference in 30-day mortality, major amputation, or overall survival between the venous and prosthetic graft groups [15].

The autologous saphenous vein is not always available for bypass, and, in those cases, either prosthetic or endovascular revascularization is an option. The incidence of an inadequate saphenous vein is reported to be 20–45% [17]. Reasons for it can be previous ablation during vein surgery, varicosis, a small-diameter vein, or previous harvest for coronary procedures.

Endovascular revascularization comprises many different techniques, much like open surgery. It can include angioplasty, different stent or stent-graft implantation, or, in the case of our study, transvenous femoropopliteal bypass with stent grafts through the deep venous system. The ZILVERPASS study recently reported their 5-year results comparing a ZILVER PTX drug-eluting stent with prosthetic open bypass for above-the-knee revascularization. This trial showed the non-inferiority of the endovascular revascularization of long TASC C and D lesions, with 1-year, 3-year, and 5-year primary patency in the endovascular group of 74.4%, 53.3%, and 49.3%, respectively [18]. Our results indicate a higher 1-year primary patency of 79.6%, but a lower 3-year primary patency of 46.2% compared to the ZILVERPASS data. Our cohort did have longer lesions (mean 27.8 cm vs. 24.7 cm). Exceptional results have been reported from Japan in a multi-center Viabahn trial. Their primary patency at 24 months for heparin-bonded stent grafts for long, complex SFA lesions was 78.8%, primary-assisted 85.7%, and secondary 92%. The mean lesion length was 21.8 cm [19]. Other authors have reported significantly lower patency rates for bare nitinol stents. Primary patency at 3 years was 32%, primary-assisted 43%, and secondary patency 53% [20]. ZILVERPASS, Viabahn, and our study results indicate remarkably higher patency rates. In an effort to expand the range of endovascular options, a novel technique has been developed to deploy stent grafts (Viabahn) through the subcutaneous tissue (in contrast to the transvenous route) in a totally percutaneous fashion. This group recently presented encouraging initial results from a cohort of 30 procedures. Their reported 3-year primary and secondary patency was 75% [21]; thus, the early results seem very promising.

Regarding the venous system, we have reported previously that transvenous endovascular bypass does not significantly impair the venous physiology and does not cause symptoms attributable to venous disease. During the follow-up, only one patient developed symptomatic DVT and was treated with anticoagulants. At the 1-year visit, the complete recanalization of occluded veins was noted. There were no significant changes in the plethysmography results or venous symptom scores during the 3-year follow-up [8].

One concerning finding that we will study further is prosthetic graft patency. It is acceptable at 12 months (72.5%) but drops substantially after that and is 22.5% at 3 years. Reasons for that could be uncorrected outflow vessel disease, poor patient selection, and compliance with post-surgical recommendations regarding medication and comorbidities. Another factor is the choice of graft. According to the literature, the saphenous vein is not available in a minority of cases. In our study, more than half of all patients considered for the control group had prosthetic bypass. The surgeon’s familiarity with the Duplex ultrasound is one of the factors that could potentially increase the proportion of venous bypasses in our center, as the availability of the saphenous vein can not be adequately assessed on computed tomography images.

There are certain limitations of this study. Most importantly, endovascular transvenous bypass was a novel and experimental procedure at the start of this study; therefore, informed consent was obviously required. As expected, a significant number of patients chose the open bypass over transvenous endovascular procedure. There is a risk of selection bias and we acknowledge it.

## 5. Conclusions

Analyzing our results, we can substantiate the universal finding that the autologous saphenous vein is the best graft to perform an above-the-knee femoropopliteal bypass. However, if the saphenous vein is not available, the patient is more obese, or open surgery is not feasible due to frailty, transvenous endovascular bypass is a viable option with comparable primary-assisted and secondary patency. Primary patency is substantially lower for endovascular transvenous compared to venous bypass. Patients treated with endovascular transvenous bypass will require a significant number of secondary procedures to provide optimal patency. Prosthetic grafts should only be used if no other option for bypass is available or technically feasible. The number of patent outflow vessels did not significantly influence patency rates.

## Figures and Tables

**Table 1:** Study population data.

	Endovascular Transvenous Group	Control Group	
Age	64.9 years (SD = 8.3)	70.6 (SD = 6.6)	*p* ≤ 0.001
Male	92.3%	70.4%	*p* ≤ 0.01
Rutherford category	3—84.6% 4—9.6% 5—5.8%	2—0.7% 3—41.9% 4—46.0% 5—11.3%	*p* ≤ 0.001
Comorbidities	Hypertension—88.4% Smoking—83.3% Coronary artery disease—28.8% Diabetes mellitus—25.9%	Hypertension—84.7% Smoking—87.8% Coronary artery disease—31.3% Diabetes mellitus—28.2%	*p* ≥ 0.5
ABI at baseline	0.62 (SD = 0.16)	0.56 (SD = 0.11)	*p* ≤ 0.001
SFA lesions length (cm, mean)	27.8 cm (SD = 4.8)	28.5 cm (SD = 5.2)	*p* ≥ 0.5
Run-off vessels	1—4/54 (7%) 2—19/54 (35%) 3—31/54 (58%)	0—2/124 (0.9%) 1—12/124 (5.5%) 2—51/124 (23.2%) 3—59/124 (26.8%)	*p* ≥ 0.5

**Table 2:** Patency data for all three groups at 12, 24, and 36 months.

Patency	Transvenous Endovascular	Venous Bypass	Prosthetic Bypass	*p* Value
**Primary**				
- 12 months	79.6%	90.9%	72.5%	0.015 vein vs. prosthetic
- 24 months	58.5%	84.1%	42.5%	<0.001 vein vs. prosthetic 0.006 vein vs. endo
- 36 months	46.2%	70.5%	22.5%	<0.05
**Primary-assisted**				
- 12 months	88.9%	93.2%	75.9%	0.013 vein vs. prosthetic
- 24 months	79.2%	86.4%	48.1%	<0.001 vein vs. prosthetic 0.002 endo vs. prosthetic
- 36 months	69.2%	77.3%	26.6%	<0.001
**Secondary**				
- 12 months	92.6%	85.2%	75.6%	0.012 vein vs. prosthetic 0.021 endo vs. prosthetic
- 24 months	86.8%	86.4%	48.7%	<0.001
- 36 months	76.9%	77.3%	28.2%	<0.001

## Data Availability

The datasets generated and analyzed during this study are available from the corresponding author upon reasonable request.
